# Three-dimensional non-approximate Coulomb interaction between two trapped quantum particles

**DOI:** 10.1038/s41598-023-45234-9

**Published:** 2023-10-24

**Authors:** Nicolás Z. Lizama, Sebastián C. Carrasco, José Rogan, Juan Alejandro Valdivia

**Affiliations:** 1 Departamento de Física, Facultad de Ciencias, Universidad de Chile, Casilla 653, 7800024 Santiago, Chile; 2 Centro para la Nanociencia y la Nanotecnolgía, CEDENNA, Santiago, 9170124 Chile; 3 Present Address: Now at the DEVCOM Army Research Laboratory, 2800 Powder Mill Road, Adelphi, MD 20783 USA

**Keywords:** Quantum mechanics, Ultracold gases

## Abstract

The two trapped quantum particles interacting problem is generalized to three dimensions, and the exact Coulomb potential is used. The system is solved by expanding the wavefunction in terms of the isotropic harmonic oscillator eigenfunctions and Hydrogen atom eigenfunctions independently, showing that each one results in a prime approximation for different domains of the normalized coupling constant of the relative interactions, suggesting that the combination of the basis is enough to build a well-suited base for the non-approximate problem. The results are compared to previous works that use a model of approximate finite-rage soft-core interaction model of the problem to give insights into the many-body states of strongly correlated ultracold bosons and fermions. We conclude that the proposed three-dimensional approach facilitates the distinction between bosons and fermions while the solutions given by the expansions better define the behavior of the particles for repulsive potentials. In addition, we discuss the substantial differences between our work and the previous approximate model.

## Introduction

Quantum control is a rapidly growing field of research developed in response to the limitations of classical control techniques, which are inadequate for controlling quantum phenomena essential for a wide range of applications, including quantum computing, quantum sensing, and quantum communication. It has played a crucial role in addressing one of the biggest challenges in quantum computing: decoherence. Decoherence limits the time a quantum system can remain in a quantum state and thus leads to errors in quantum computing. By developing controlling techniques, researchers have been able to extend the coherence time of qubits, enabling more complex computations to be performed. Ultra-cold atoms provide an excellent platform for studying and implementing these quantum-controlling techniques. Because of their low temperatures and well-defined quantum states, ultra-cold atoms can be manipulated and controlled with high precision using external fields. Thanks to the advances in technology in the last two decades, which allow not only to create a system of ultra-cold atoms but to manipulate each particle and control the interaction between them with great accuracy^1–4^, the study of trapped quantum particles has blossomed. Problems previously considered only of academic and theoretical nature are now the basis of several emergent quantum technologies.

The two-body system represents one of the most fundamental frameworks for interacting system of trapped atoms and the building foundation of few- and many-body physics^1, 2, 5, 6^, which has been studied in various ways^7–16^ in the past. However, most of these works approximate the interaction by replacing the Coulomb potential with simpler choices to try to construct exact analytical solutions. For example, a quasi-exact solution to the Schrödinger equation for two interacting particles was obtained by Busch *et al.*^9^ using a delta-like interaction, a model that was later extended to Gaussian-shaped potentials^17^. Analytical solutions for more complex interactions, like dipole-dipole, are increasingly needed because of the limitations observed^15, 18^. For example, depending on the length scale or the size of the trap, one should study and use different considerations, like the van der Waals interaction for short scale, Coulomb corrections^19^, s-wave scattering length characterizing low-energy atomic collisions, or atomic collisions for traps whose size is comparable to the size associated with the atomic interaction^20^.

In addition, the study of quantum particles trapped in a harmonic oscillator represents a useful approximation of the microsystems trapped in an optical lattice, so a precise understanding of the interactions in this elementary model may help comprehend the physics occurring in more complex and realistic systems. For instance, stationary solutions are helpful to study the non-equilibrium dynamic of two atoms^21–24^. This is true with all other simplified models previously cited and those before Kościk and Sowiński^7^, whose work is used as the primary reference in this paper. In the spirit of keep developing new simplified models from which we could build more complex physics, Kościk et al. proposed a finite-rage soft-core interaction model of two trapped interacting quantum particles as an approximation of the Coulomb potential and many properties of the system were derived. Similar to the comparison they made with Busch et al. ^9^, mainly focusing on the spectral properties of the system, we will aim to enlarge the already vast pool of studies made in this area but focusing not on the quasi-exact solution, but on devising a proper base to numerically construct solutions to the non-approximate interaction between two particles trapped in a harmonic oscillator.

Hence, we present the generalization of this problem by considering the numerical solution to the exact Coulomb interaction potential in the diagonalization of the relative motion Hamiltonian between the two trapped particles. To do so, we use the three-dimensional isotropic harmonic oscillator eigenfunctions to expand the Hamiltonian and solve the eigenproblem, studying the system behavior when considering the combined harmonic and hydrogen-atom systems. Two distinguishable domains are identified and a clear suggestion on how to build the Hilbert space for a more in detail study is deduced: a combination of the harmonic eigenfunctions, which work nicely for the limit of a repulsive interaction; and Hydrogen atom eigenfunctions, which provide a good approximation for an attractive interaction. A proper combination might be enough to build a well-suited base for the exact Coulomb potential problem between these limits.

## Model

The first step we take to generalize the work of Kościk and Sowiński^7^ is to consider three dimensions, so we have a system described by the following Hamiltonian:

1 $$\begin{aligned} \mathscr {H}&= -\frac{\hbar ^2}{2m}\left( \nabla _1^2 + \nabla _2^2 \right) + \frac{m\omega ^2}{2}(x_1^2+x_2^2) + V(|\textbf{x}_2-\textbf{x}_1|) \end{aligned}$$

where *V* corresponds to the Coulomb potential. We use boldface to indicate vectors and normal font to refer to their norm. Using the standard transformation to the coordinates of the center of mass:

$$\begin{aligned}&\textbf{r} = \textbf{x}_2 - \textbf{x}_1,~~~\textbf{R} = \frac{\textbf{x}_1 + \textbf{x}_2}{2}, \end{aligned}$$

it is possible to separate Eq. (1) as the sum of two independent Hamiltonians $\mathscr {H} = \mathscr {H}_R + \mathscr {H}_r$, one corresponding to the center of mass ($\textbf{R}$) and the other to the relative distance between particles ($\textbf{r}$), which have the following form:

2 $$\begin{aligned} \mathscr {H}_R&= -\frac{\hbar ^2}{4m}\nabla ^2_R + m\omega ^2 R^2~, \end{aligned}$$

3 $$\begin{aligned} \mathscr {H}_r&= -\frac{\hbar ^2}{2\mu }\nabla ^2_r + \frac{\mu }{2}\omega ^2 r^2 + \frac{ Z_1 Z_2 e^2}{r},~ \end{aligned}$$

where $\mu$ is the reduced mass. While $\mathscr {H}_R$ has the known form of the isotropic quantum harmonic oscillator (IHO), $\mathscr {H}_r$ has an extra interaction given by the Coulomb potential. The main focus of this report is to find a well-suited base to solve the eigenproblem given by Eq. (3) and, therefore, have a better understanding of the exact Coulomb interaction between quantum particles in a trap. To do so, we consider the dimensionless operators:

$$\begin{aligned} \hat{p} = \sqrt{\frac{1}{\mu \hbar \omega }} ~p,&\hspace{2cm} \hat{r} = \sqrt{ \frac{\mu \omega }{\hbar } }~r, \end{aligned}$$

with the momentum operator $p=-i\hbar \nabla_r$, to transform Eq. (3) into the dimensionless Hamiltonian

4 $$\begin{aligned} \mathscr {H}_r = \hbar \omega \left[ \frac{1}{\hat{r}^2}\frac{\partial}{\partial \hat{r}}\left( \hat{r}^2 \frac{\partial}{\partial \hat{r}} \right) + \frac{\hat{r}^2}{2} - \frac{L^2}{2 \hat{r}^2} + \sqrt{ \frac{\mu }{\hbar ^3 \omega } } ~\frac{Z_1 Z_2 e^2}{\hat{r}}\right] ~, \end{aligned}$$

where $L^2$ is the magnitude of the angular momentum operator, such that the *spherical harmonic* functions are eigenfunctions of $L^2$, namely, $L^2 Y_{\ell ,m}(\theta ,\phi )=-\ell (\ell +1) Y_{\ell ,m}(\theta ,\phi )$. Notice that the model only has one free parameter $\gamma = Z_1 Z_2 e^2\sqrt{\mu /h^3\omega }$, unlike the finite-range interaction of Ref.^7^, where a strength interaction parameter *V* and a range parameter *a* that describes the distance in which the particles interact with each other are needed. In our case, as we do not approximate the Coulomb interaction, we consider the range parameter $a\rightarrow \infty$ and only need $\gamma$ to help us study the intensity of attraction ($\gamma <0$) or repulsion ($\gamma >0$) between the two particles. Furthermore, using $\gamma =0$ and $\gamma \rightarrow - \infty$ recovers the IHO and a Hydrogen-like atom, respectively, which we use to compare later.

## Method

As $\mathscr {H}_r$ may suggest, we approach this problem using two different known solutions as a base: the three-dimensional isotropic harmonic oscillator and the Hydrogen-like atom (HA). We take one of these basis functions and expand the “extra” term in Eq. (4) to build the complete matrix we later diagonalize. For example, if we use the IHO eigenfunctions, we end up with a diagonal matrix given by the eigenenergies of the Harmonic problem plus a matrix corresponding to the Coulomb term, which we build expanding $\gamma /r$ on the IHO base. Considering the Hydrogen eigenfunctions, we would have a diagonal matrix given by the Hydrogen energies plus a harmonic matrix expanded into the HA base. Having this in mind, we only need to calculate the extra term in both scenarios to diagonalize and find the system’s spectra.

Starting with the known solution to the three-dimensional IHO:

5 $$\begin{aligned} \Psi _{n\ell m}(r,\theta ,\phi )&= r^\ell f_{n\ell }(r) Y_{\ell m}(\theta ,\phi )e^{-\mu \omega r^2/2\hbar } ~, \end{aligned}$$

where $f_{n\ell }$ is a polynomial with coefficients given by the recurrent relationship

6 $$\begin{aligned} (q+2)(q + 2\ell + 3) \, a_{q+2}&= \frac{2\mu \omega }{\hbar }\left( q - n \right) a_q ~. \end{aligned}$$

This solution already lets us distinguish the symmetric and anti-symmetric spatial wave function with respect to an exchange of particles positions, given by the symmetry of the spherical harmonics:

7 $$\begin{aligned} Y_{\ell m}(\pi -\theta ,\phi +\pi )&= (-1)^\ell Y_{\ell m}(\theta ,\phi ). \end{aligned}$$

This is a simple distinction: an even value of $\ell$ corresponds to bosons and an odd value of $\ell$ to fermions. We then have a natural separation between particle types because of the three-dimensional solution we have chosen.

Continuing with the eigenproblem, we end up with the following equation using (5) and (4)

8 $$\begin{aligned} \langle \Psi _{n,\ell }|\mathscr {H}_r|\Psi _{n',\ell '}\rangle&= E^{\text {IHO}}_{nn' \ell \ell '} \delta _{nn'}\delta _{\ell \ell '} + \langle \Psi _{n,\ell }|\gamma /r|\Psi _{n',\ell '}\rangle , \end{aligned}$$

and since the energy levels of the harmonic oscillator are

9 $$\begin{aligned} E^{\text {IHO}}_{n,\ell }&= \left( n + \ell + \frac{3}{2} \right) \hbar \omega , \end{aligned}$$

we only need to calculate the matrix elements of the Coulomb interaction, which can be reduced to expressions involving the Gamma function

10 $$\begin{aligned} \langle \Psi _{n,\ell }|\gamma /r|\Psi _{n',\ell '}\rangle&= \frac{\gamma }{2}\sum _{\begin{array}{c} q=0\\ j=0 \end{array}}^{N} a_q ~a_j \beta ^{-(q+j+\ell +\ell '+2)/2} ~\Gamma \left( \frac{q+j+\ell +\ell '+2}{2}\right) ~, \end{aligned}$$

with $$\beta =\mu \omega /\hbar$$.

Conversely, using the radial part of the HA eigenfunctions

11 $$\begin{aligned} \varphi _{n\ell m}(r,\theta ,\phi ) = R_{n\ell }(r)Y^m_\ell (\theta ,\phi ), \end{aligned}$$

we end up with a new equation similar to Eq. (8), namely,

12 $$\begin{aligned} \langle \varphi _{n\ell }|\mathscr {H}_r|\varphi _{n'\ell '}\rangle&= E^{\text {HA}}_{nn' \ell \ell '} \delta _{nn'}\delta _{\ell \ell '} + \frac{\mu \omega ^2}{2}\langle \varphi _{n\ell }| r^2 |\varphi _{n'\ell '}\rangle . \end{aligned}$$

Equations (8) and (12) summarize the eigenproblem we will focus on since they provide the non-diagonal matrices to test the efficiency and accuracy when using either the IHO or the HA eigenbasis in the solution via expansion of two trapped quantum particles interacting via a non-approximate Coulomb potential. The spectral properties and the shape of the orbitals obtained using this method will be compared to the numerical solution of the Schrödinger equation with the relative motion Hamiltonian (4) using the finite elements method (FEM), in which we discretize $\mathscr {H}\psi (r)=E_{\ell }\psi (r)$ into a mesh using divided differences with a spatial discretization $\Delta r$ to transform it into a matrix eigenvalue problem. Using standard techniques we obtain the eigenstates $\psi _{n,\ell }(r)$ with eigenenergies $E_{n,\ell }$.

## Results

For simplicity, we will analyze the case of particles with equal mass, so $\mu = m/2$. We use energies in dimensionless units and normalize the distance with respect to the Bohr radius ($a_0$). We obtain an efficient convergence of the energies of a repulsive ($0<\gamma$) system $H_r$ using the IHO eigenfunctions with $\ell =0$ when solving the eigenproblem given by Eq. (8). In fact, we obtain a good approximation to the ground state with just five elements in the base. This tendency continues as we calculate higher energies: we only need $n+3$ to $n+5$ elements in the IHO base for the *n*-th energy value to converge. For example, when going from $n = 5$ to $n = 10$, with $\ell =0$ and $\gamma = 5$, the relative error in the 5-th eigenvalue decreases by 38%, from 17.4 to 12.6.

We then contrast these results using second-order perturbation theory to check that the approximation converges to the proper result. A further check is made by rearranging the Hamiltonian to obtain the known Hydrogen atom energy levels as follows: we have

$$\begin{aligned} \mathscr {H}_r&= \mathscr {H}_{IHO} + V_1 \end{aligned}$$

if we consider $\gamma =-1$ such that $V_1 = -1/r$, then the HA Hamiltonian is

13 $$\begin{aligned} \mathscr {H}_{HA}&= \mathscr {H}_r - V_2 \nonumber \\&=\mathscr {H}_{IHO} + V_1 - V_2, \end{aligned}$$

with $V_2$ as the harmonic potential. Solving Eq. (13) with the same expansion used for the IHO eigenfunctions results in a suitable approximation for the known Hydrogen atom energies $E^{\text {HA}}_n = -\frac{1}{2n^2}$ in atomic units, where different values for the amplitude of oscillation gives us additional precision. Therefore we confirm that the IHO eigenfunctions build a valuable basis for this system.

On the other hand, calculating the matrix elements with HA eigenfunctions to solve the diagonalization problem with the expansion of the harmonic potential, produces, as expected, a poor result as HA eigenfunctions do not form a complete basis if we do not consider the scattering states: Hydrogen atom eigenfunctions on their own, despite converging, does not work and results in wrong values for the system energy levels.

**Figure 1:**
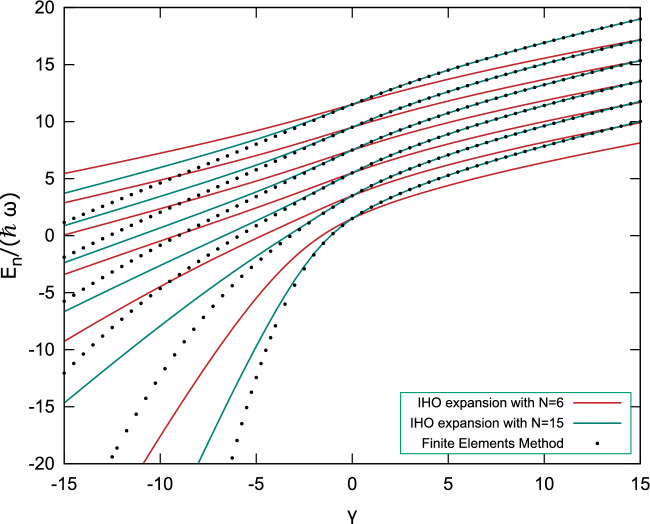
Spectrum of the normalized Hamiltonian (4) as a function of the coupling constant $\gamma$ using the first 6 (red) and 15 (teal) eigenstates of the IHO base to expand and solve the eigenproblem. These results are compared to the direct solution of the differential equation via the finite elements method (dotted) with $\Delta r=0.01$, showing that, as the number of elements used to solve the diagonalization problem increases, the energy gets closer to those obtained by the finite elements method.

**Figure 2:**
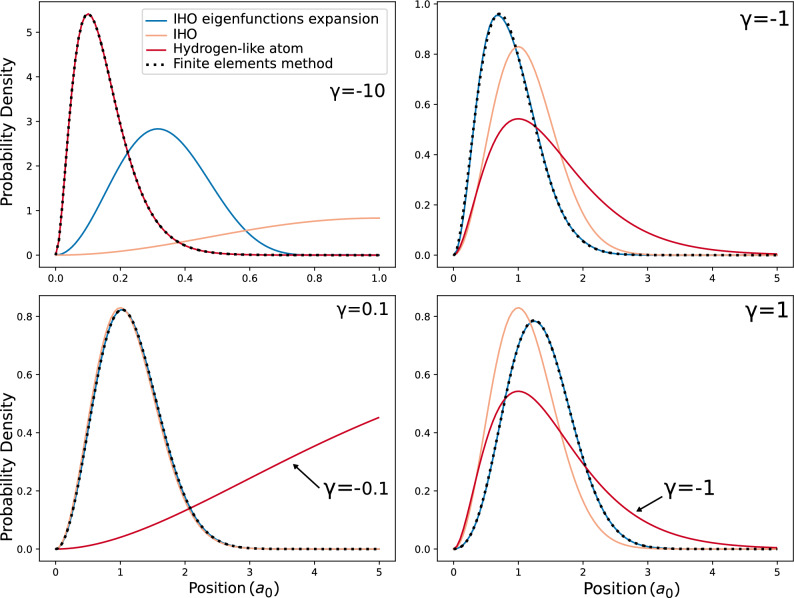
Radial probability density distribution of the relative motion in bosonic ground state $(n,\ell )=(0,0)$ for different values of the attraction coefficient $\gamma$, where $a_{0}$ is the Bohr radius. As a repulsive potential does not allow the Hydrogen-like eigenfunctions to be localized, we use attractive potentials when $\gamma =0.1$ and $\gamma =1$. It can be seen that our solution (blue) using 18 elements of the IHO base best approximates the numerical solution (dotted) for $0<\gamma$, where the system approaches the IHO (soft orange). For $\gamma \ll 0$, where the system resembles the hydrogen-like (red) behavior, our method fails to model the system, just like Fig. 1 shows.

A further study of the behavior of the system is made by analyzing how the energy levels change by the coupling factor $\gamma$ in Eq. (4) and comparing them to those obtained by Kościk and Sowiński, and the direct solution to the differential equation via the finite elements method, which can be seen in Fig. 1. The finite element solution was calculated using divided differences up to 2nd order for the derivatives, using $\Delta r=0.01$. For the calculation, we write the eigenvalue equation in terms of finite elements up to $r_{max}=10$ in normalized units with a $\Delta r$ resolution. In Fig. 2, we note that this is a reasonable value of $r_{max}$ from the trapped system. In addition, we studied the eigenvalue convergence for other values of $r_{max}$ and reached the conclusion that $r_{max}=10$ was a reasonable compromise between calculation time and accuracy. We also checked with $\Delta r=0.001$, and we obtained the same result as in Fig. 1. Additionally, the use of isotropic harmonic oscillator eigenfunctions with $\ell =0$ results in an excellent approximation for the repulsive potential $0<\gamma$ while failing for $\gamma \ll 0$, which represents a highly attractive potential between the particles. For a broader analysis of this behavior, we contrast the radial probability density distribution of the IHO, HA, the system solved using 18 IHO eigenfunctions and finite elements method for different values of $\gamma$, as shown in Fig. 2. We can see that, opposite to the IHO, the HA base reasonably describes the attractive system $\gamma < 0$ while failing to approximate the repulsive case $0 < \gamma$. For this last case, we used $\gamma =-0.1$ and $\gamma =-1$ to calculate the Hydrogen-like density distribution while using repulsive potentials for the other three curves. An analogous analysis was made by plotting a set of three-dimensional orbitals of the Hydrogen atom and comparing them to those obtained by solving the relative motion Hamiltonian (4), which are shown in Table 1. Despite the differences in scale, which are informed by the expectation value of the radius, it can be seen that the solution of the relative motion Hamiltonian for a highly attractive potential results in a shape identical to that of the Hydrogen atom.

**Figure 3:**
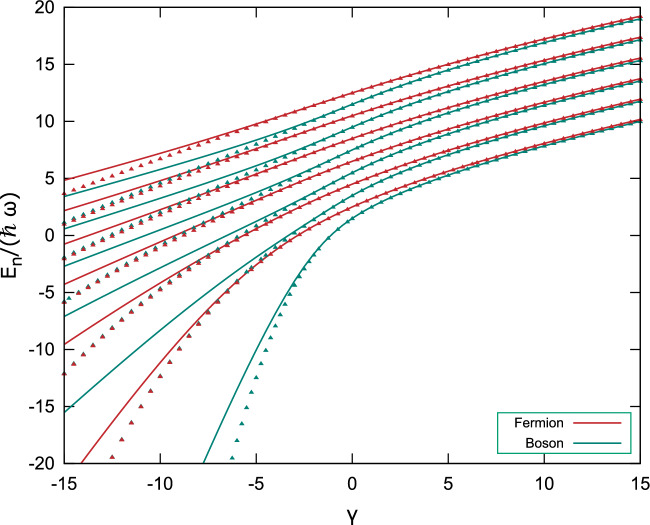
Spectrum of the relative motion Hamiltonian $\mathscr {H}_r$ as a function of the coupling factor $\gamma$ for bosonic $(n,\ell )=(0,0)$ (teal) and fermionic $(n,\ell )=(0,1)$ (red) ground state obtained using 18 elements in the expansion in IHO eigenfunctions (solid). These results are compared to the FEM solution (triangles) using $\Delta r = 0.01$. There is a more accurate behavior between neighboring eigenenergies for the repulsive non-approximated interaction, as the degeneracy between them should be obtained in the hard-core limit $\gamma \rightarrow \infty$.

Finally, as we mention in Eq. (7), the distinction between bosons and fermions is reduced to the parity of $\ell$, so using $\ell =1$ helps us describe the interaction between two fermions, whose contrast with a bosonic $(n,\ell )=(0,0)$ system can be seen in Fig. 3. Despite getting closer for positive values of $\gamma$, the two systems are not equal as previous works^7^ have suggested. Moreover, the exact solution using FEM shows that there is also degeneracy between neighboring bosonic and fermionic energy levels for the highly attractive potential. Another meaningful difference between symmetric and antisymmetric particles is that the fermion eigenfunction converges faster to the expected result due to the Pauli exclusion principle.

## Conclusions and Discussion

We focused on the non-approximate Coulomb interaction between two trapped particles using the isotropic harmonic oscillator and Hydrogen atom eigenfunctions independently to expand the relative motion Hamiltonian between two trapped quantum particles and numerically solve the eigenproblem by diagonalization, showing that each of these functions has complementary domains in which they result in a good approximation to the system. The IHO eigenfunctions are an ideal base for the repulsive potential but also work for weak attraction, resulting in adequate approximations for values up to $\gamma \approx -5$, as shown in Fig. 1. In contrast, the HA eigenfunctions work best for highly attractive potentials. This behavior was visualized by studying the radial density distribution of our model and comparing it to the isotropic harmonic oscillator and the Hydrogen atom, as well as the numerical solution obtained by the finite elements method. The system’s inclination towards the two different domains is shown in Fig. 2, where it is clear that the IHO basis fails to approximate the system when it approaches the Hydrogen-like atom. Furthermore, the similarity to the Hydrogen atom for highly attractive potentials $\gamma \ll 0$ is checked in Table 1 at the $\gamma =-10$ column; despite the differences in scale due to the harmonic trap, it is identical to the Hydrogen atom orbitals. This suggests that a combination of these functions may be enough to build a base that helps us solve the non-approximate Coulomb interaction between trapped particles using the expansion in eigenfunctions. The wide domain in which IHO functions work and the low number of elements that are needed in the base for the approximation tells us that maybe a low number of HA eigenfunctions are required to be added for the solution to be generalized to positive and negative values of $\gamma$, thus solving the differences observed in Figs. 1 and 2 between our results and the numerical result using FEM.

As we think that the expansion in the IHO basis may be useful for further studies, we include the coefficients of the IHO expansion for a number of $\gamma$ values as supplementary data.

Our model, which has just one variable coefficient to explore the attraction and repulsion between trapped particles, captures qualitatively the results from previous works and deepens the understanding of what features are specific to the election of a particular potential. Thus it expands on the problem that started with Busch et al. In addition, our analysis clearly distinguishes between the behavior of bosons and fermions, a characterization that comes naturally with a three-dimensional approach. With the exact potential, substantial differences are observed in degeneracy for bosonic and fermionic energy levels: degeneracy is observed between neighboring eigenenergies for highly attractive systems, as Fig. 3 shows; and degeneracy for coefficients $0<\gamma$ more accurately approach the hard-core limit. This makes us confident that with few additions, a robust model can be built to later study different types of interaction and other numbers of particles, thus adding more theoretical understanding to an area at the forefront of experimental studies.

**Table 1:**
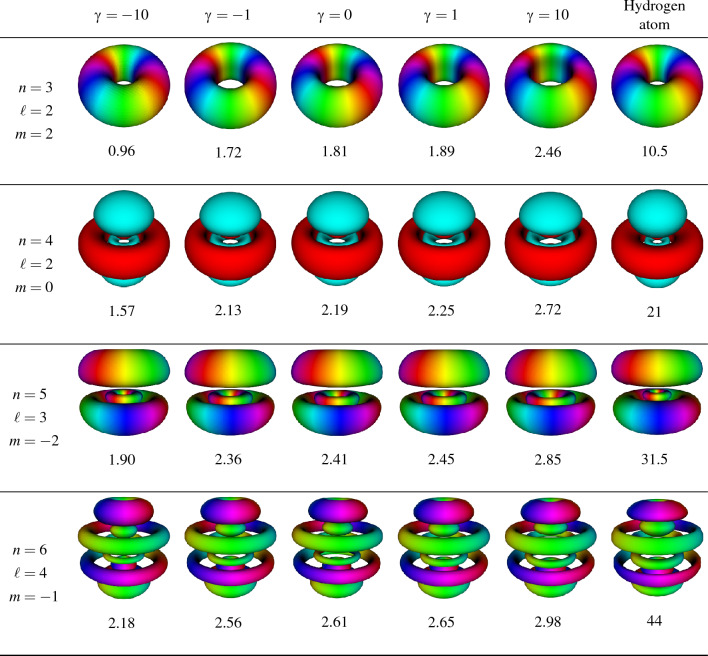
Three-dimensional visualization of the orbitals of the Hydrogen atom compared to those obtained by solving the relative motion Hamiltonian (4) for attractive and repulsive coupling constants $\gamma$ (columns) and different atomic numbers (rows). As Eq. (7) states, odd (even) values of $\ell$ correspond to fermionic (bosonic) particles. The expectation value for radius is displayed under every figure to show the difference in scale. This is mainly due to the effect of the harmonic trap but also because of the attractive potential in the case of $\gamma =-10,-1$. Despite the similarities at first glance, details in the geometry and size of the orbitals change, where it can be seen that the highly attractive system approaches the Hydrogen atom orbital best.

### Supplementary Information

Supplementary Information 1.

Supplementary Information 2.

## Data Availability

Data generated during this study are included in this published article and its supplementary information files.
